# The Regulation of Glutamate Transporter 1 in the Rapid Antidepressant-Like Effect of Ketamine in Mice

**DOI:** 10.3389/fnbeh.2022.789524

**Published:** 2022-03-02

**Authors:** Yaping Chen, Mengxin Shen, Xu Liu, Jiangping Xu, Chuang Wang

**Affiliations:** ^1^Zhejiang Provincial Key Laboratory of Pathophysiology, Ningbo University School of Medicine, Ningbo, China; ^2^School of Pharmaceutical Sciences, Southern Medical University, Guangzhou, China; ^3^College of Pharmacy, Fujian University of Traditional Chinese Medicine, Fuzhou, China; ^4^Department of Pharmacy, General Hospital of Chinese People’s Armed Police Forces, Beijing, China

**Keywords:** glutamate transporter 1, depression, ketamine, α-Amino-3-hydroxy-5-methyl-4-isoxazolepropionic acid receptor, L-type voltage-dependent calcium channel

## Abstract

Accumulating evidence suggests that glutamate clearance plays a critical role in the pathophysiology and treatment of depression. Preclinical and clinical studies have demonstrated that ketamine provides an immediate and sustained antidepressant effect. However, the precise mechanism of its action remains to be elucidated. Glutamate transporter 1 (GLT1) participates in glutamate clearance; therefore, we hypothesized that GLT1 may play an important role in the antidepressant effect of ketamine. In this study, we determined that GLT1 inhibition blocks the antidepressant-like properties of ketamine and alters the phosphorylation of the mammalian target of rapamycin (mTOR) in the prefrontal cortex (PFC). Our results show that pretreatment with dihydrokainic acid (DHK), a GLT1 inhibitor, alleviated the antidepressant-like effect of ketamine, and decreased the level of phosphorylated mTOR (pmTOR) in mice (which is normally upregulated by ketamine). In addition, inhibition of α-amino-3-hydroxy-5-methyl-4-isoxazole-propionic acid (AMPA) receptor and L-type voltage-dependent calcium channel (L-VDCC) significantly abolished the antidepressant-like effect of ketamine. Moreover, inhibition of L-VDCC significantly blocked the upregulation of GLT1 and BDNF in the PFC of mice. The inhibition of the AMPA receptor only significantly alleviated BDNF. Our results provide insight into the role of GLT1 as the critical presynaptic molecule participating in the pathophysiological mechanism of depression and contributing to the antidepressant-like effect of ketamine. In addition, our study confirms that both AMPA receptor and L-VDCC are crucial factors in the immediate antidepressant-like effect of ketamine.

## Introduction

Depression is one of the most prevalent psychiatric disorders, characterized by high incidence and treatment resistance. However, currently available antidepressants have several major drawbacks, such as low response rates and delayed therapeutic effects (Gaynes et al., 2009). The pathophysiology of depression as well as targets of pharmacological treatments have been defined by the monoamine hypothesis of depression for the last decades. Antidepressants aiming at these previously defined targets normally require from 3 to 8 weeks to produce a therapeutic response; however, they affect neurotransmitters immediately (Machado-Vieira et al., 2008). A single sub-anesthetic dose of ketamine, a glutamate N-methyl-D-aspartate (NMDA) receptor antagonist, based on two meta-analyses of randomized placebo-controlled trials (Fond et al., 2014; McGirr et al., 2015), meets the needs of a rapid-acting antidepressant treatment. However, the mechanism underlying this immediate antidepressant effect in animal models remains largely unknown (Kavalali and Monteggia, 2015).

Ketamine has been described as a powerful antagonist of NMDA receptors, however, researchers have shown that the mechanisms underlying this response are likely to be more complex than a selective blockade of NMDA receptors (Naughton et al., 2014; Chaki and Fukumoto, 2015). Glutamatergic systems have been found to play an important role in the rapid antidepressant effect of ketamine (Krystal et al., 2013; Lener et al., 2017; Machado-Vieira et al., 2017). Glial cells regulate glutamatergic systems by clearing glutamate from extracellular space via excitatory amino acid transporter (EAAT). The glutamate is then recycled in the glutamate-glutamine cycle. Without the activity of glutamate transporters, glutamate builds up and kills cells in a process called excitotoxicity. It has been reported that β-lactam antibiotic ceftriaxone increases the uptake of glutamate by upregulating the expression of EAATs, and therefore exerts neuroprotective (Rothstein et al., 2005) and antidepressant effects (Mineur et al., 2007). There are five subtypes of EAATs in humans, as well as rodents. Subtypes EAAT1-2 are found in the membranes of glial cells (Lehre et al., 1995). EAAT2, also known as glutamate transporter 1 (GLT1), is responsible for over 90% of glutamate reuptake in the central nervous system (CNS) (Matsugami et al., 2006; Holmseth et al., 2009; Rao et al., 2015). GLT1 has been reported to play a critical role in the antidepressant effect of ketamine, and its mechanism of action may be associated with brain-derived neurotrophic factor (BDNF)/tropomyosin receptor kinase B (TrkB) signaling (Liu et al., 2016). However, the upstream regulatory mechanism by which GLT1 participates in the antidepressant effect of ketamine remains to be clarified.

In the present study, we examined the effects of GLT1 on the rapid antidepressant-like effect of ketamine in chronic unpredictable mild stress (CUMS) mice and explored the pathways that may participate in the regulation of GLT1 in the prefrontal cortex (PFC) of mice. First, we inhibited the activity of GLT1 with dihydrokainic acid (DHK) in CUMS mice. We then determined the level of mammalian target of rapamycin (mTOR), which is responsible for the rapid action of ketamine (Jernigan et al., 2011). Furthermore, we aimed to clarify the roles of the α-amino-3-hydroxy-5-methyl-4-isoxazolepropionic acid receptor (AMPAR) and L-type voltage-dependent calcium channel (L-VDCC) in the regulation of GLT1. These two proteins participate in the upstream pathways of BDNF as well as TrkB, and have been reported to play an important role in the rapid antidepressant-like effects of ketamine (Duman et al., 2012). Additionally, BDNF and TrkB activation regulated by AMPAR and L-VDCC are required for the rapid antidepressant effects of ketamine (Autry et al., 2011), and BDNF is a potent endogenous activator of mTOR, which has also been suggested to underlie the antidepressant action of ketamine (Li et al., 2010).

## Materials and Methods

### Animals and Drugs

The adult male C57BL/6J mice (6–8 weeks) used for the experiment were supplied by the Laboratory Animal Center of the Southern Medical University (Guangzhou, China). The animals were housed in an air-conditioned room at 22 ± 3°C and 60 ± 5% relative humidity under a 12 h light/12 h dark cycle (lights on at 7:00 a.m.) with *ad libitum* access to food and water. All experiments were carried out in accordance with the principles of the “NIH Guide for the Care and Use of Laboratory Animals” (NIH Publications No. 80–23, revised 1996). The procedures were approved by the Animal Care and Use Committee of the Southern Medical University. Ketamine hydrochloride, NBQX, and DHK used in this work were obtained from Sigma (St. Louis, MO or Shanghai, China). Verapamil was purchased from Aladdin Ltd., (Shanghai, China). All of them were dissolved in saline to the required concentration.

### Experimental Design and Drug Treatment

**FIGURE 1:**
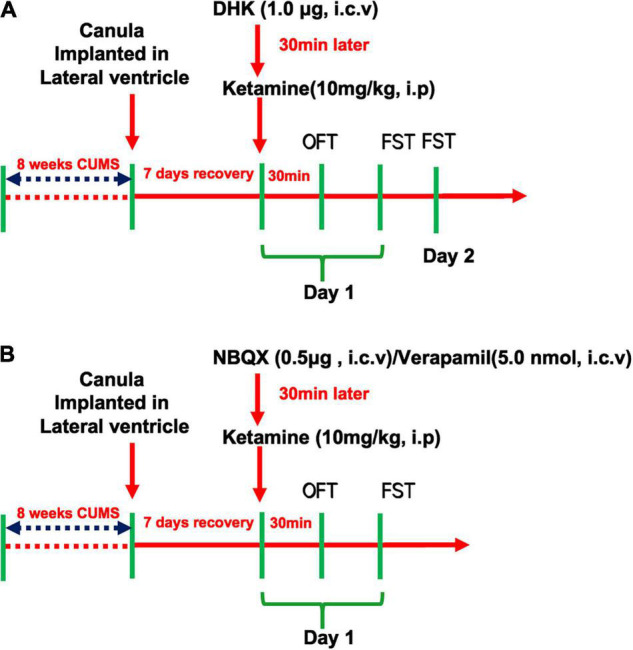
Schedule of drug treatment and behavioral tasks orders. The experiment design of GLT1 inhibition in the rapid antidepressant-like effect of Ketamine **(A)** and the involvement of AMPAR/L-VDCC in the regulation of Ketamine on GLT1 **(B)**.

The first aim of the present study was to explore the role of GLT1 in ketamine-induced rapid-acting antidepressant-like effects in CUMS mice. The selective inhibitor of GLT-1, DHK (1.0 μg), or vehicle was microinjected by intracerebroventricular (i.c.v.) injection in mice. Intraperitoneal injection of ketamine (10 mg/kg, i.p) was administered 30 min after the DHK treatment, and then the open field test (OFT) and forced swim test (FST) were conducted successively in the light phase between 8:00 a.m. and 4:00 p.m. in day 1 and 2 (as shown in Figure 1A).

In addition, to assess the roles of AMPAR and L-VDCC in the antidepressant-like actions of ketamine, the NBQX (0.5 μg, i.c.v) and Verapamil (5.0 nmol, i.c.v) were pretreatment 30 min before the ketamine administration, respectively, in mice. And then the OFT and FST were conducted successively in the same day (as shown in Figure 1B).

### Surgery for Brain Cannula Implantation and Drug Injections

Intracerebroventricular cannulation implantation was performed as described (Li et al., 2017). The stereotaxic coordinates for the lateral ventricle were carried out in accordance with the Paxinos/Franklin mouse atlas (Paxinos and Franklin, 2001). All mice were anesthetized with 2% isoflurane and mounted on a stereotactic frame (R WD Kopf Instruments, Shenzhen, China) with a Kopf model mouse adaptor. Stainless steel guide cannula were implanted in the lateral ventricle at 0.70 mm posterior to the bregma, 1.30 mm lateral to the midline, and 2.00 mm below the skull surface. The guide cannula was anchored to the skull with dental cement and a stainless-steel stylet was inserted to maintain patency for microinjection. Animals were housed individually and allowed 7 days for recovery.

During the microinfusion, the mice were indisposed with a gaseous anesthetic and woke up 10∼20 min after infusion, and could be applied to behavior tests to explore the rapid antidepressant-like effects of ketamine in mice. The infusions were performed via a 10-μl Hamilton microsyringe connected to the microinfusion cannula via 0.26-mm ID polyethylene tubing. Microinfusions were carried out over 5 min with an infusion pump at 1 μL/min, and the cannulas were left in place for 3 additional minutes to avoid backflow. To verify the correct placement of the cannula after i.c.v drug delivery, mice were sacrificed after behavioral tests and cryostat sections of lateral ventricle cut through the cortex determine the cannula track. Only animals with the correct cannula placement were used for further analysis. Mice were sacrificed by dislocated spine method under anesthesia (ether), the tissues used for western blot analysis were collected 6 h after ketamine treatment.

### Chronic Unpredictable Mild Stress Procedure

This animal model of stress consists of chronic exposure to variable unpredictable stressors, none of which is sufficient alone to induce long-lasting effects. Briefly, the CUMS procedure (Huang et al., 2017) involved 12 different stressors that were randomly arranged throughout the day and night over 56 consecutive days. The stressors were (1) 24 h of food deprivation, (2) 1 h of exposure to 4°C room (3) 24 h of exposure to a 45° cage tilt, (4) overnight illumination, (5) 24 h of exposure to a wet cage (100 ml of water per individual cage, which is enough to make the sawdust bedding wet), (6) 5 min of swimming in 6∼8°C water, (7) tail clamp for 5 min, (8) 24 h of water deprivation, (9) unpredictable shocks for 5 min (15 mA, one shock/5, 10 s duration), (10) Swimming for 15 min, (11) 4 h of restricted movement, and (12) 4 h of disrupting the cage. The behavioral tests were performed and scored by trained and experienced observers who were blinded to the animals’ conditions.

### Open Field Test

Briefly, the Open Field Test (OFT) was conducted according to the previous protocols that we recently reported (Li et al., 2017; Lv et al., 2018). The 50 × 50-cm arena with 39-cm high walls is made of a white Plexiglas box. Two black lines were drawn on the floor. Mice were placed into the center of the arena and allowed to explore the apparatus for 5 min. The number of the line crossings and rearings were considered parameters of locomotor activity and recorded over a 5-min period by a digital system.

### Forced Swimming Test

The FST was conducted in a sound-attenuated room according to the previous studies with minor changes (Wu et al., 2016; Zanos et al., 2016; Lv et al., 2018). Briefly, mice were placed individually for 6 min into a clear plastic cylinder (diameter 10 cm, height 25 cm) containing 10 cm of fresh water, maintained at 23 ± 2°C. The immobility time was recorded over the following 4 min of the 6-min testing duration. The immobility time was defined as time when a mouse floated with only the bare minimum activity necessary to keep their heads above the water.

### Western Blot Analysis

Frozen PFC tissues in each group (*n* = 3) of mice were homogenized in ice-cold radio-immunoprecipitation assay (RIPA) lysis buffer containing protease and phosphatase inhibitors cocktail (Pierce Biotechnology, Rockford, IL, United States). Lysates were centrifuged at 12,000 × g for 30 min at 4°C. The protein concentration of each sample lysate was determined with the BCA kit (Thermo Scientific, Rockford, IL). Each sample (25 μg total protein) was separated on 10% SDS polyacrylamide gel electrophoresis (PAGE) gels and transferred to PVDF membranes (0.22 μm; Millipore, CA). Membranes were then incubated with anti-mTOR (1:1,000, Cell Signaling), anti-phospho-mTOR (1:1,000, Cell Signaling), anti-GLT1 (1:1,000, Santa Cruze), BDNF (1:800, Abcam, United States) and anti-GAPDH (1:2,000, Millipore, CA) at 4°C overnight. The membranes were then incubated with Alexa Fluor800-conjugated antibody (1:10,000, Invitrogen, Eugene, OR) for 60 min. Target bands were captured with the fluorescence scanner (Odyssey Infrared Imaging System, LI-COR Biotechnology, Lincoln, NE) and quantified with Image J.

### Statistical Analysis

Data are presented as mean ± standard error of the mean (SEM). Statistical analysis of the data was performed using one-way analysis of variance (ANOVA) followed by Tukey’s *post-hoc* test as appropriate using GraphPad Prism software (Version 5.0, Prism software for PC, GraphPad). Values of *P* < 0.05 were considered statistically significant.

## Results

### Role of Glutamate Transporter 1 in the Antidepressant-Like Effects of Ketamine in Chronic Unpredictable Mild Stress Mice

**FIGURE 2:**
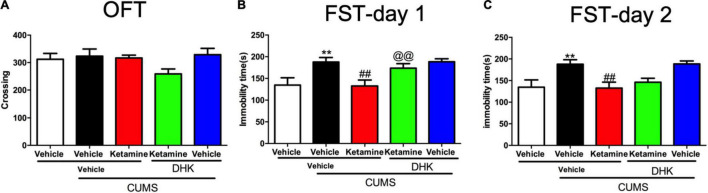
The influence of DHK on the rapid antidepressant effect of Ketamine. The crossing of mice in OFT **(A)**; the immobility time of mice in day 1 **(B)**; the immobility time of mice in day 2 **(C)**. The data are expressed as means ± SEM, *n* = 10–12. ^**^P < 0.01 compared with vehicle + vehicle + non-stressed group; ^##^*P* < 0.01 compared with vehicle + vehicle + CUMS group; ^@@^*P* < 0.01 compared with ketamine + vehicle + CUMS group.

The CUMS exposure did not alter the locomotor activities of the mice in OFT [*F*(4, 51) = 1.712, *P* = 0.1616, Figure 2A]. While, the immobility time of FST was significantly increased by CUMS in day 1 [*F*(4, 51) = 5.228, *P* < 0.01, Figure 2B] and day 2 [*F*(4, 51) = 5.447, *P* < 0.01, Figure 2C]. A single administration of ketamine (10 mg/kg) significantly reversed these two alterations in CUMS procedure (both *P* < 0.01). However, the decrease of immobility time produced by ketamine was only significantly blocked by intracerebroventricular (i.c.v.) injection of DHK on day 1 (*P* < 0.01, Figure 2B), suggesting that GLT1 plays a critical role in the rapid-acting antidepressant-like effects of ketamine in mice. We did not find the antidepressant-like actions of ketamine was abolished by DHK 24 h after infusion in the FST (Day 2, Figure 2C). As shown in Supplementary Figure 1, the different doses of ketamine (3, 10, and 30 mg/kg, i.p) did not significantly change the locomotor activities. However, these Three doses of ketamine produced significant antidepressant-like actions in the FST and TST of mice (Supplementary Figure 2).

### Ketamine Activated Mammalian Target of Rapamycin in the Prefrontal Cortex of Chronic Unpredictable Mild Stress Mice and Was Significantly Alleviated by Dihydrokainic Acid

**FIGURE 3:**
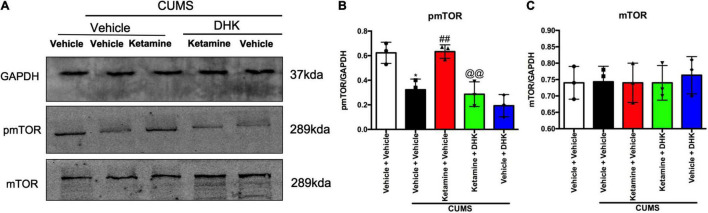
The influence of DHK on the mTOR phosphorylation in the PFC of mice. mTOR and pmTOR protein expression were determined by Western blot. Representative western blot **(A)** and quantification **(B,C)** of fold changes in the protein levels of pmTOR and mTOR in the PFC of mice. The data are expressed as means ± SEM; *n* = 3; *P < 0.05 compared with vehicle + vehicle + non-stressed group; ^##^*P* < 0.01 compared with vehicle + vehicle + CUMS group; ^@@^*P* < 0.01 compared with ketamine + vehicle + CUMS group.

To investigate whether the activation of mTOR produced by ketamine in an animal model of depression was abolished by DHK, the expressions of phosphorylation of mTOR (pmTOR) and total mTOR were determined in the PFC of mice (Figure 3A). As shown in Figure 3B, the CUMS exposure resulted in a significant decrease in protein levels of pmTOR when compared with the control group (*P* < 0.05). In comparison with the CUMS group, ketamine treatment significantly elevated pmTOR protein expression (*P* < 0.01). However, pretreatment with DHK, the up-regulation on the ratio of pmTOR in the PFC of mice produced by ketamine was significantly abolished (*P* < 0.01). Figure 3C showed the changes of mTOR, there was no significant change among all groups [*F*(4, 10) = 0.1079, *P* = 0.9770].

### The Involvement of α-Amino-3-Hydroxy-5-Methyl-4-Isoxazolepropionic Acid Receptor and L-Type Voltage-Dependent Calcium Channel in the Rapid-Acting Antidepressant-Like Effects of Ketamine in the Prefrontal Cortex of Mice

**FIGURE 4:**
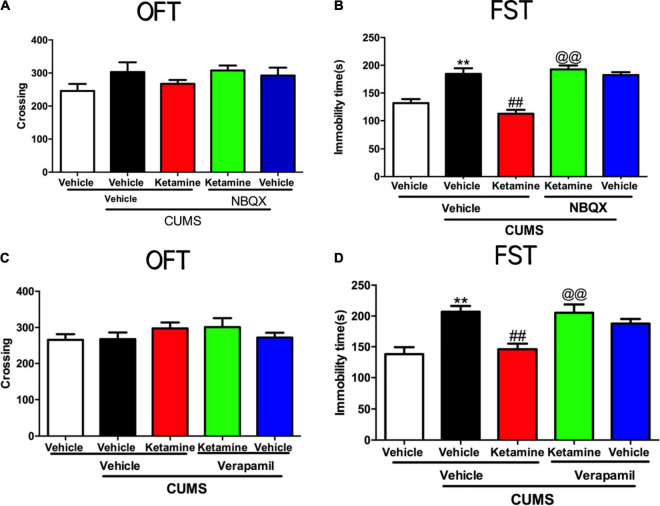
The roles of AMPAR and L-VDCC played in the rapid antidepressant effect of Ketamine. The crossing of mice in an open field test in AMPAR blocked **(A)** and L-VDCC inhibition **(C)**; the immobility time of mice in forced swimming test in AMPAR blocked **(B)** and L-VDCC inhibition **(D)**. The data are expressed as means ± SEM, *n* = 10. ^**^P < 0.01 compared with vehicle + vehicle + non-stressed group; ^##^*P* < 0.01 compared with vehicle + vehicle + CUMS group; ^@@^*P* < 0.01 compared with ketamine + vehicle + CUMS group.

Given the growing evidence that AMPAR and L-VDCC may involve in the rapid-acting antidepressants (Duman et al., 2012; Lepack et al., 2014; Zanos et al., 2016), we aimed to explore whether the GLT1 and BDNF were regulated by AMPAR and L-VDCC in the antidepressant-like actions of ketamine in mice. As shown in Figures 4A,C, the locomotor activities of the mice were not changed in the line crossings [NBQX, *F*(4, 45) = 1.523, *P* = 0.2115, Figure 4A; Verapamil, *F*(4, 45) = 0.8936, *P* = 0.4757, Figure 4C] of OFT by all treatments. However, both pretreatment with NBQX [*F*(4, 45) = 22.56, *P* < 0.001, Figure 4B] and verapamil [*F*(4, 45) = 10.03, *P* < 0.001, Figure 4D] can completely reversed the antidepressant-like effects of ketamine in the FST of mice. As shown in Supplementary Figure 3. Single treatment with NBQX (microinjection) and verapamil (i.p.) had no effects alone in the OFT and FST of mice.

### The Different Role of α-Amino-3-Hydroxy-5-Methyl-4-Isoxazolepropionic Acid Receptor and L-Type Voltage-Dependent Calcium Channel in the Regulation on the Glutamate Transporter 1 by Ketamine in the Prefrontal Cortex of Mice

**FIGURE 5:**
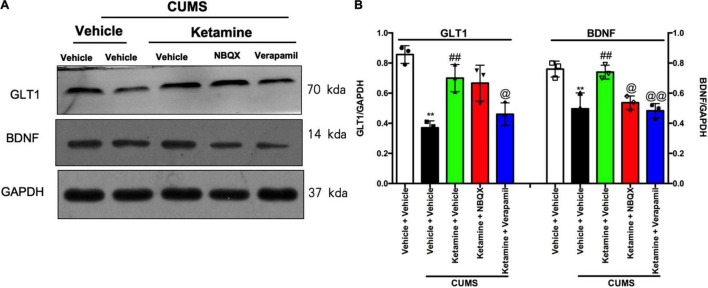
The roles of AMPAR and L-VDCC in the regulation of Ketamine on GLT1 and BDNF in the PFC of mice. GLT1 and BDNF protein expression were determined by Western blot. Representative western blot (A) and quantification (B) of fold changes in the protein levels of GLT1, and BDNF in the PFC of mice. The data are expressed as means ± SEM; *n* = 3; **P < 0.01 compared with vehicle + vehicle + non-stressed group; ^##^*P* < 0.01 compared with vehicle + vehicle + CUMS group; ^@^*P* < 0.05, ^@@^*P* < 0.01 compared with ketamine + vehicle + CUMS group.

Growing evidence has shown that the AMPAR and L-VDCC are activated by rapid-acting antidepressants, resulting in the fast release of BDNF and activation of downstream pathways (Lepack et al., 2014; Yao et al., 2017; Yu et al., 2017, 2018; Ghosal et al., 2018). To evaluate the roles of AMPAR and L-VDCC in the regulation of GLT1 and BDNF by ketamine, we analyzed levels of the GLT1 and BDNF by western blot analysis in the PFC of mice (Figure 5A). After 56 days of CUMS exposure, the levels of GLT1 [*F*(4, 10) = 17.01, *P* = 0.0002, Figure 5B] and BDNF [*F*(4, 10) = 13.63, *P* = 0.0005, Figure 5B] were significantly decreased in the PFC of mice. However, a single treatment with ketamine rapidly and significantly reversed these molecular changes (GLT1, *P* < 0.01; BDNF, *P* < 0.01, Figure 5B). In contrast, the levels of BDNF (NBQX, *P* < 0.05; verapamil, *P* < 0.001) in the PFC of mice were significantly abolished by pretreatment with verapamil, respectively. Notably, the up-regulation on the GLT1 of ketamine was significantly alleviated by verapamil in the PFC of mice (*P* < 0.05). Interestingly, pretreatment with NBQX, the up-regulation on the GLT1 by ketamine was not significantly abolished compared with single treatment with ketamine in the PFC of mice.

## Discussion

A variety of studies in patients have shown that glutamatergic and rodent models dysregulation is involved in depression (Li et al., 2019). GLT1 is responsible for the majority (90%) of extracellular and synaptic glutamate clearance in the CNS. Previous studies have suggested that a decrease in the level of GLT1 in the brain is a possible cause of depression (Cui et al., 2014; Rappeneau et al., 2016). Specifically, infusion of DHK (GLT1 inhibitor) into the brain has been shown to alter the levels of amino acids (Fallgren and Paulsen, 1996) and induce both anxiety and depressive-like symptoms (John et al., 2015; Gasull-Camos et al., 2017a). In the above reports, DHK exhibited its effects within 5–15 min, and biological assays and behavior tests were completed in 24 h, similar to the behavior tests in our study. GLT1 levels in the PFC corresponding to the effect after 48 h of DHK infusion were established by western blot analyses.

In our experiments, GLT1 expression levels in the PFC were significantly decreased following CUMS stimulation and returned to normal by ketamine treatment, which was consistent with the results of previous studies (Choudary et al., 2005; Liu et al., 2016). GLT1 inhibition is known to induce depression-like behaviors (Bechtholt-Gompf et al., 2010), whereas upregulation of GLT1 can have an antidepressant effect (Rothstein et al., 2005; Sanacora et al., 2007; Bechtholt-Gompf et al., 2010; Ding et al., 2017). Our current work confirmed that pretreatment with the GLT1 inhibitor DHK significantly alleviated the rapid antidepressant-like effect of ketamine infusion, which was consistent with the results of previous studies (Choudary et al., 2005; Liu et al., 2016). The decrease in astrocytic Glu uptake due to the decreased level of GLT1 might lead to a shortage of Gln in astrocytes, which is responsible for the release of Glu into the presynaptic neuron, which may support our findings. Our findings may be also confirmed by previous studies in which the infusion of DHK into the brain has been shown to change the levels of amino acids (Fallgren and Paulsen, 1996) and induce both anxiety and depressive-like symptoms (John et al., 2015; Gasull-Camos et al., 2017a).

Activation of the mTOR signaling pathway has been recently shown to be a critical factor in the antidepressant effect of ketamine (Hoeffer and Klann, 2010). The rapid induction of mTOR phosphorylation occurs within 30 min of ketamine administration. This, in turn, leads to the activation of mTOR-dependent protein synthesis (Duman et al., 2012). Here, we assessed changes in the level of the phosphorylated mTOR (pmTOR) after DHK treatment and found that the downregulation of GLT1 significantly inhibited the regulatory effect of ketamine on pmTOR. Therefore, it can be concluded that the regulatory action of ketamine on GLT1 influences mTOR activity. GLT1 as the major glial glutamate transporter is located in the membranes of pre-synaptic astrocytes and is responsible for more than 90% of glutamate uptake.

Glutamate is an important excitatory neurotransmitter in the CNS. This molecule as well as its cognate receptors have been described as new targets for rapid antidepressant action (Dutta et al., 2015; Machado-Vieira et al., 2017). Here, we speculated that GLT1 indirectly influences the level of mTOR phosphorylation through the regulation of glutamate levels during the rapid antidepressant effect of ketamine.

AMPAR and L-VDCC have both been demonstrated to play important roles in the rapid antidepressant effect of ketamine as one of the critical downstream fast-acting factors. This finding has been confirmed by behavior tests in the current study. We also measured BDNF expression levels and found that ketamine regulated the levels of BDNF in CUMS mice. Both NBQX and verapamil pretreatment abolished the effects of ketamine on BDNF, consistent with previous reports (Jourdi et al., 2009; Lepack et al., 2014; Zhou et al., 2014). It is likely that L-VDCC may play a key role in the regulatory effect of ketamine on GLT1. However, the effect of L-VDCC inhibition on the level of GLT1 expression was different from that of AMPAR inhibition. AMPAR antagonist treatment increased GLT1 expression. We believe that this effect may be due to the participation of AMPA in the serotonergic activity in the PFC. It was reported that DHK and S-AMPA microinfusion in IL evoked similar antidepressant-like effects in the FST at 10 min post-administration (Gasull-Camos et al., 2018). Moreover, the GLT1 inhibition has been reported to induce a rapid increase in serotonergic activity in IL, which was blocked by NBQX (Gasull-Camos et al., 2017b). However, in our experiments we only detected the total GLT1 expressed in the PFC (not IL); therefore, further research is needed to clarify the changes in GLT1 expression in the IL after AMPA inhibition. The signaling pathway induced by the communication between glial and neuronal cells is often difficult to study. Data suggest that astrocytes signal to neurons through the Ca^2+^-dependent release of glutamate (Haydon and Carmignoto, 2006; Fiacco et al., 2009). Additionally, our biochemical studies revealed that the calcium channel blocker verapamil significantly inhibited the effect of ketamine in CUMS mice.

This may be evidence of the influence of calcium channels on GLT1 expression. Growing evidence suggests that the antidepressant-like effects of ketamine and scopolamine in rodent models are caused by an influx of extracellular glutamate, elevated levels of BDNF, and activation of L-VDCC (Lepack et al., 2014; Wohleb et al., 2017; Ghosal et al., 2018; Yu et al., 2018). This may explain why the regulation of GLT1 by ketamine was blocked by t e calcium channel antagonist verapamil.

This study has some limitations. We found that GLT1 expression changed in both the hippocampus and PFC in mice under CUMS. As the PFC is an integral region in the top-down regulation of behavior and control of stress reactivity (Arnsten, 2015), we studied the biological changes in this area as a representation of the brain region. Accumulating evidence indicates that the hippocampus and nucleus accumbens (NAc) are also involved in depression-like phenotypes (Tsankova et al., 2006; Zhang et al., 2014; Yang et al., 2015). Thus, it is necessary to clarify the changes in these areas in future studies.

## Conclusion

In conclusion, we showed that the antidepressant-like effect of ketamine on CUMS mice was prevented by GLT1 inhibition and that the regulation of mTOR phosphorylation in the PFC of mice affected the action of GLT1. Our results also indicated that L-VDCC in the PFC may influence the regulatory role of GLT1 in the therapeutic mechanism of ketamine.

## Data Availability Statement

The raw data supporting the conclusions of this article will be made available by the authors, without undue reservation.

## Ethics Statement

The animal study was reviewed and approved by the Animal Care and Use Committee of the Southern Medical University.

## Author Contributions

YC, MS, XL, JX, and CW performed the experiments and article preparation. CW wrote the first draft of the manuscript, which all other authors reviewed. All authors approved publication.

## Conflict of Interest

The authors declare that the research was conducted in the absence of any commercial or financial relationships that could be construed as a potential conflict of interest.

## Publisher’s Note

All claims expressed in this article are solely those of the authors and do not necessarily represent those of their affiliated organizations, or those of the publisher, the editors and the reviewers. Any product that may be evaluated in this article, or claim that may be made by its manufacturer, is not guaranteed or endorsed by the publisher.
