# Supplementary figures and images for: The Regulation of Glutamate Transporter 1 in the Rapid Antidepressant-Like Effect of Ketamine in Mice
Source: Front Behav Neurosci. 2022 Mar 2;16:789524. doi: 10.3389/fnbeh.2022.789524 (PMC8926310; doi:10.3389/fnbeh.2022.789524)

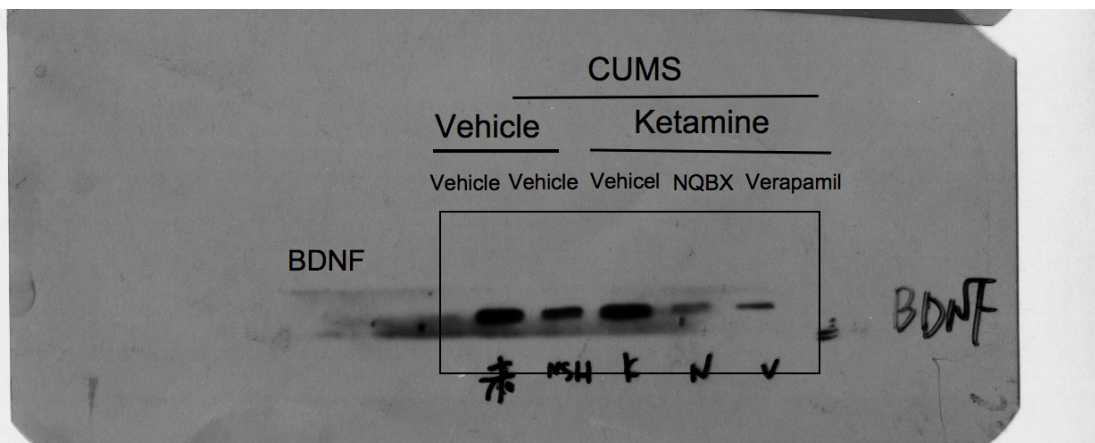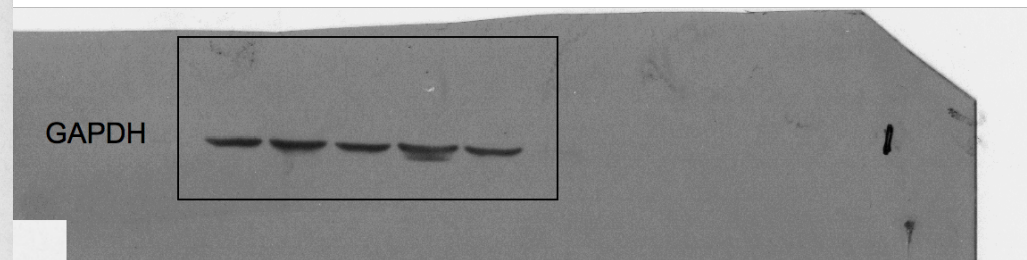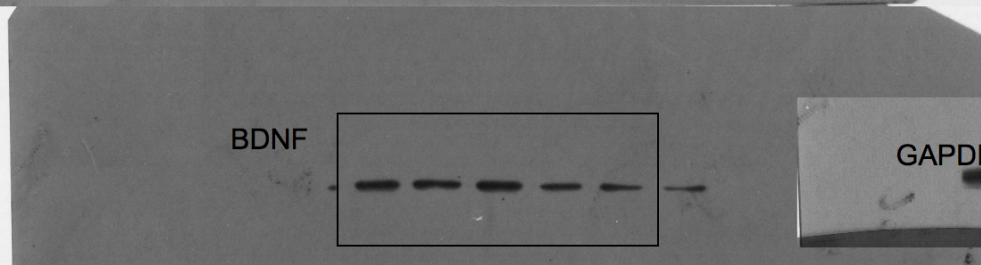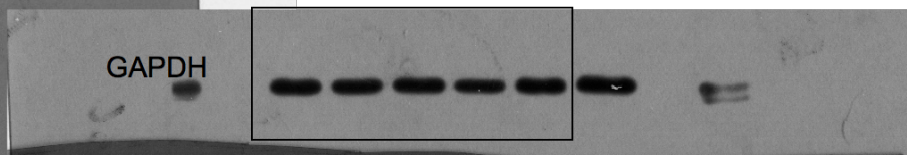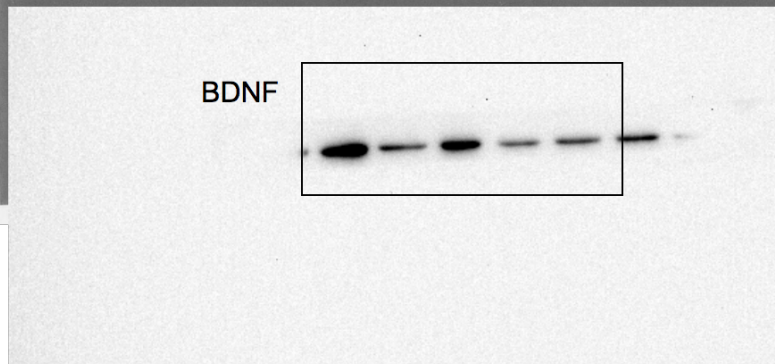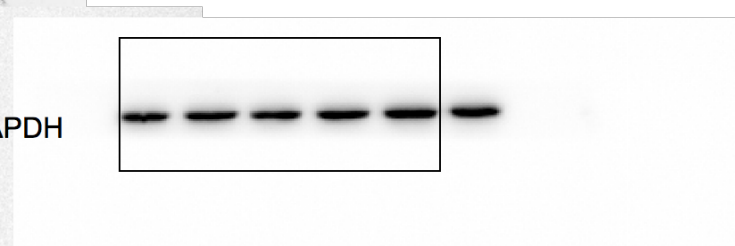

Supplement: Supplementary file 1 [file Data_Sheet_1.PDF]

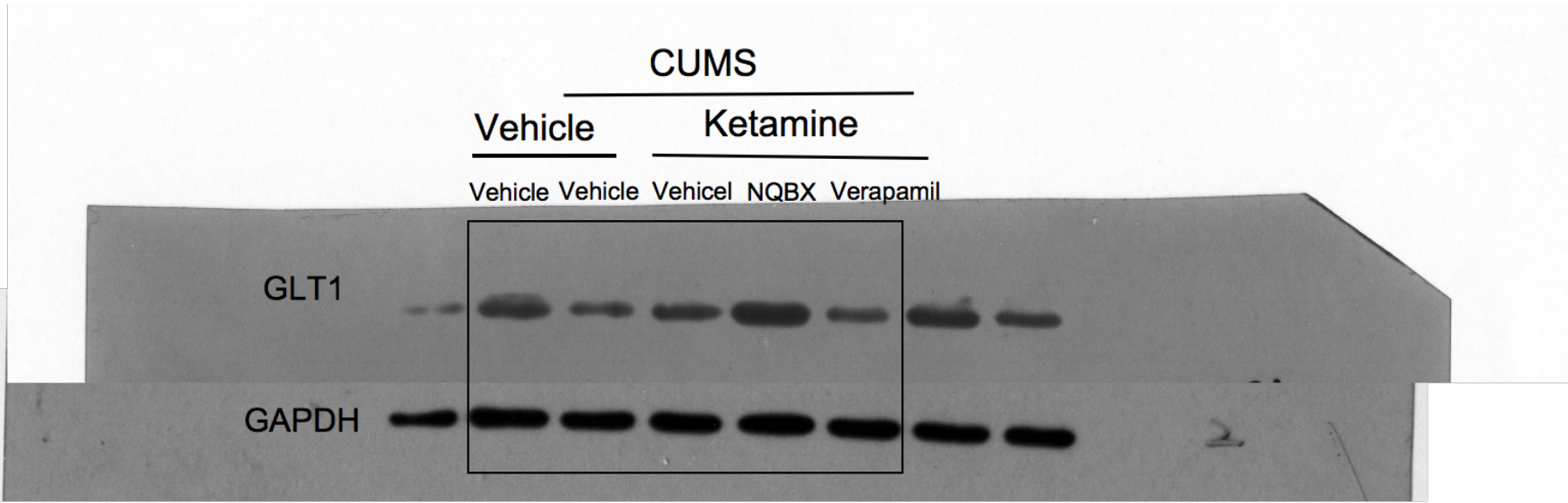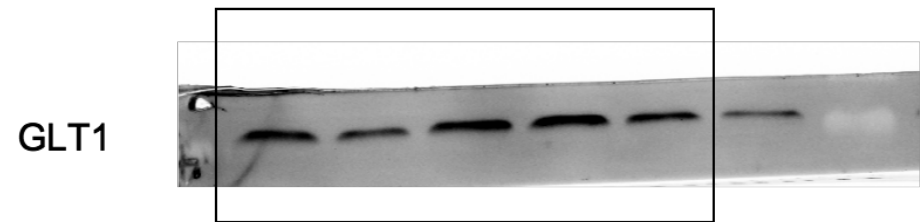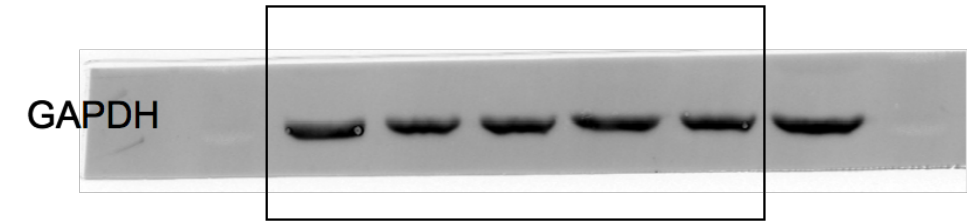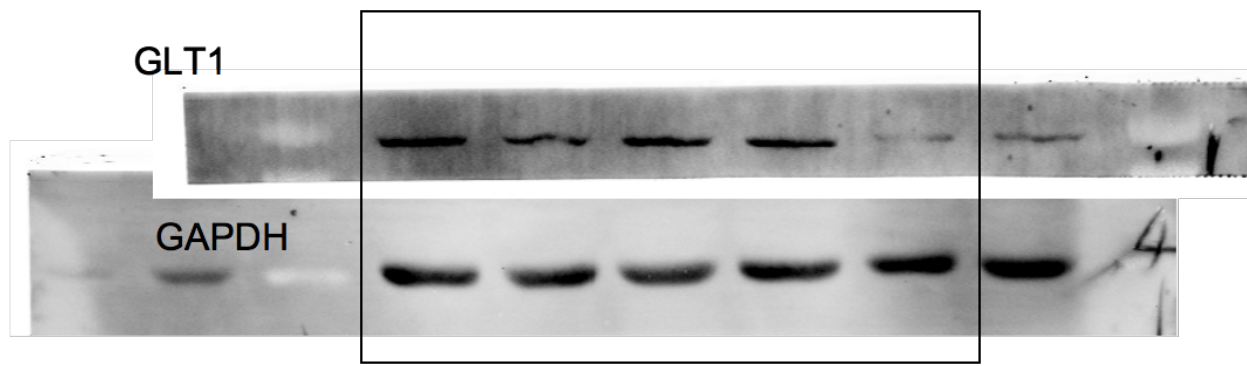

Supplement: Supplementary file 2 [file Data_Sheet_2.PDF]
