# Supplementary material for: The Regulation of Glutamate Transporter 1 in the Rapid Antidepressant-Like Effect of Ketamine in Mice
Source: Front Behav Neurosci. 2022 Mar 2;16:789524. doi: 10.3389/fnbeh.2022.789524 (PMC8926310; doi:10.3389/fnbeh.2022.789524)
Supplement: Supplementary file 3 [file Data_Sheet_3.PDF]

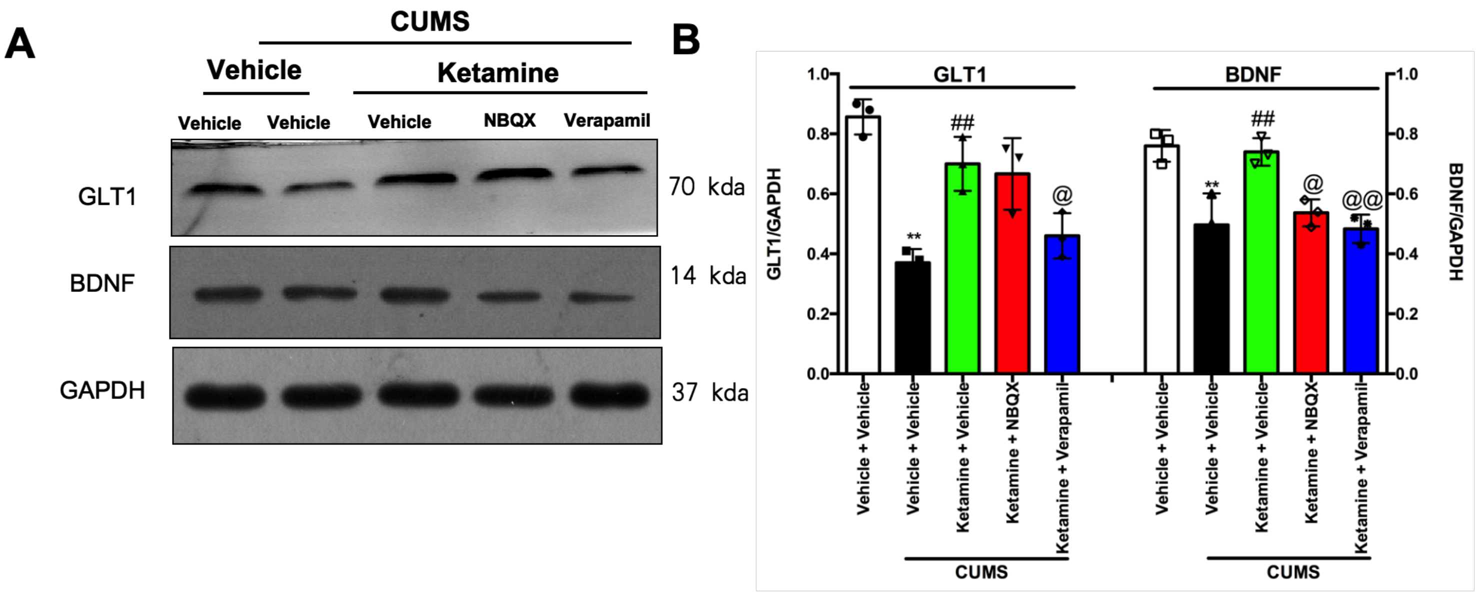

	Ordinary one-way ANOVA<br>ANOVA	A	B	C	D	E
		Data Set-A	Data Set-B	Data Set-C	Data Set-D	Data Set-E
		Y	Y	Y	Y	Y
1	Table Analyzed	GLT1 BDNF				
2						
3	ANOVA summary					
4	F	17.01				
5	P value	0.0002				
6	P value summary	***				
7	Are differences among means statistically significant? (P < 0.05)	Yes				
8	R square	0.8719				
9						
10	Brown-Forsythe test					
11	F (DFn, DFd)	0.2495 (4, 10)				
12	P value	0.9034				
13	P value summary	ns				
14	Significantly different standard deviations? (P < 0.05)	No				
15						
16	Bartlett's test					
17	Bartlett's statistic (corrected)					
18	P value					
19	P value summary					
20	Significantly different standard deviations? (P < 0.05)					
21						
22	ANOVA table	SS	DF	MS	F (DFn, DFd)	P value
23	Treatment (between columns)	0.4568	4	0.1142	F (4, 10) = 17.01	P = 0.0002
24	Residual (within columns)	0.06713	10	0.006713		
25	Total	0.5239	14			
26						
27	Model comparison	SS	DF	Probability it is correct		
28	Null H. All population means identical	0.5239	14	0.13%		
29	Alternative H: Distinct population means	0.06713	10	99.87%		
30	Ratio of probabilities			780.2		
31	Difference in AICc			13.32		
32						
33	Data summary					
34	Number of treatments (columns)	5				
35	Number of values (total)	15				

	Ordinary one-way ANOVA<br>ANOVA	A	B	C	D	E
		Data Set-A	Data Set-B	Data Set-C	Data Set-D	Data Set-E
		Y	Y	Y	Y	Y
1	Table Analyzed	GLT1 BDNF				
2						
3	ANOVA summary					
4	F	13.63				
5	P value	0.0005				
6	P value summary	***				
7	Are differences among means statistically significant? (P < 0.05)	Yes				
8	R square	0.8451				
9						
10	Brown-Forsythe test					
11	F (DFn, DFd)	0.5560 (4, 10)				
12	P value	0.6997				
13	P value summary	ns				
14	Significantly different standard deviations? (P < 0.05)	No				
15						
16	Bartlett's test					
17	Bartlett's statistic (corrected)					
18	P value					
19	P value summary					
20	Significantly different standard deviations? (P < 0.05)					
21						
22	ANOVA table	SS	DF	MS	F (DFn, DFd)	P value
23	Treatment (between columns)	0.2203	4	0.05508	F (4, 10) = 13.63	P = 0.0005
24	Residual (within columns)	0.04040	10	0.004040		
25	Total	0.2607	14			
26						
27	Model comparison	SS	DF	Probability it is correct		
28	Null H. All population means identical	0.2607	14	0.53%		
29	Alternative H: Distinct population means	0.04040	10	99.47%		
30	Ratio of probabilities			187.7		
31	Difference in AICc			10.47		
32						
33	Data summary					
34	Number of treatments (columns)	5				
35	Number of values (total)	15				
