# Supplementary material for: The Regulation of Glutamate Transporter 1 in the Rapid Antidepressant-Like Effect of Ketamine in Mice
Source: Front Behav Neurosci. 2022 Mar 2;16:789524. doi: 10.3389/fnbeh.2022.789524 (PMC8926310; doi:10.3389/fnbeh.2022.789524)
Supplement: Supplementary file 4 [file Data_Sheet_4.PDF]

Ordinary one-way ANOVA<br>ANOVA		A<br>Data Set-A<br>Y	B<br>Data Set-B<br>Y	C<br>Data Set-C<br>Y	D<br>Data Set-D<br>Y	E<br>Data Set-E<br>Y
1	Table Analyzed	Data 1				
2						
3	ANOVA summary					
4	F	1.523				
5	P value	0.2115				
6	P value summary	ns				
7	Are differences among means statistically significant? (P < 0.05)	No				
8	R square	0.1193				
9						
10	Brown-Forsythe test					
11	F (DFn, DFd)	1.079 (4, 45)				
12	P value	0.3782				
13	P value summary	ns				
14	Significantly different standard deviations? (P < 0.05)	No				
15						
16	Bartlett's test					
17	Bartlett's statistic (corrected)	8.488				
18	P value	0.0752				
19	P value summary	ns				
20	Significantly different standard deviations? (P < 0.05)	No				
21						
22	ANOVA table	SS	DF	MS	F (DFn, DFd)	P value
23	Treatment (between columns)	27311	4	6828	F (4, 45) = 1.523	P = 0.2115
24	Residual (within columns)	201691	45	4482		
25	Total	229002	49			
26						
27	Data summary					
28	Number of treatments (columns)	5				
29	Number of values (total)	50				

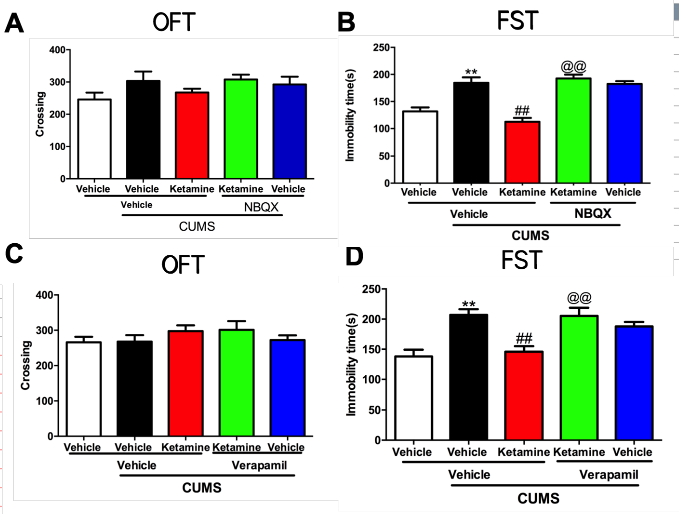

Ordinary one-way ANOVA<br>ANOVA		A<br>Data Set-A<br>Y	B<br>Data Set-B<br>Y	C<br>Data Set-C<br>Y	D<br>Data Set-D<br>Y	E<br>Data Set-E<br>Y
2						
3	ANOVA summary					
4	F	22.56				
5	P value	< 0.0001				
6	P value summary	****				
7	Are differences among means statistically significant? (P < 0.05)	Yes				
8	R square	0.6673				
9						
10	Brown-Forsythe test					
11	F (DFn, DFd)	0.2228 (4, 45)				
12	P value	0.9243				
13	P value summary	ns				
14	Significantly different standard deviations? (P < 0.05)	No				
15						
16	Bartlett's test					
17	Bartlett's statistic (corrected)	3.937				
18	P value	0.4146				
19	P value summary	ns				
20	Significantly different standard deviations? (P < 0.05)	No				
21						
22	ANOVA table	SS	DF	MS	F (DFn, DFd)	P value
23	Treatment (between columns)	52165	4	13041	F (4, 45) = 22.56	P < 0.0001
24	Residual (within columns)	26009	45	578.0		
25	Total	78174	49			
26						
27	Model comparison	SS	DF	Probability it is correct		
28	Null H. All population means identical	78174	49	0.00%		
29	Alternative H: Distinct population means	26009	45	100.00%		
30	Ratio of probabilities			0.0		
31	Difference in AICc			45.33		
32						
33	Data summary					
34	Number of treatments (columns)	5				
35	Number of values (total)	50				

Ordinary one-way ANOVA<br>ANOVA		A<br>Data Set-A<br>Y	B<br>Data Set-B<br>Y	C<br>Data Set-C<br>Y	D<br>Data Set-D<br>Y	E<br>Data Set-E<br>Y
1	Table Analyzed	Data 1				
2						
3	ANOVA summary					
4	F	0.8936				
5	P value	0.4757				
6	P value summary	ns				
7	Are differences among means statistically significant? (P < 0.05)	No				
8	R square	0.07359				
9						
10	Brown-Forsythe test					
11	F (DFn, DFd)	0.2554 (4, 45)				
12	P value	0.9049				
13	P value summary	ns				
14	Significantly different standard deviations? (P < 0.05)	No				
15						
16	Bartlett's test					
17	Bartlett's statistic (corrected)	3.833				
18	P value	0.4291				
19	P value summary	ns				
20	Significantly different standard deviations? (P < 0.05)	No				
21						
22	ANOVA table	SS	DF	MS	F (DFn, DFd)	P value
23	Treatment (between columns)	11700	4	2925	F (4, 45) = 0.8936	P = 0.4757
24	Residual (within columns)	147295	45	3273		
25	Total	158995	49			
26						
27	Data summary					
28	Number of treatments (columns)	5				
29	Number of values (total)	50				

Ordinary one-way ANOVA<br>ANOVA		A<br>Data Set-A<br>Y	B<br>Data Set-B<br>Y	C<br>Data Set-C<br>Y	D<br>Data Set-D<br>Y	E<br>Data Set-E<br>Y
1	Table Analyzed	Verapamil				
2						
3	ANOVA summary					
4	F	10.03				
5	P value	< 0.0001				
6	P value summary	****				
7	Are differences among means statistically significant? (P < 0.05)	Yes				
8	R square	0.4713				
9						
10	Brown-Forsythe test					
11	F (DFn, DFd)	0.4946 (4, 45)				
12	P value	0.7397				
13	P value summary	ns				
14	Significantly different standard deviations? (P < 0.05)	No				
15						
16	Bartlett's test					
17	Bartlett's statistic (corrected)	3.500				
18	P value	0.4779				
19	P value summary	ns				
20	Significantly different standard deviations? (P < 0.05)	No				
21						
22	ANOVA table	SS	DF	MS	F (DFn, DFd)	P value
23	Treatment (between columns)	42913	4	10728	F (4, 45) = 10.03	P < 0.0001
24	Residual (within columns)	48144	45	1070		
25	Total	91057	49			
26						
27	Model comparison	SS	DF	Probability it is correct		
28	Null H. All population means identical	91057	49	0.00%		
29	Alternative H: Distinct population means	48144	45	100.00%		
30	Ratio of probabilities			0.0		
31	Difference in AICc			22.17		
32						
33	Data summary					
34	Number of treatments (columns)	5				
[illegible]	[illegible]	[illegible]				
