# Supplementary material for: The Regulation of Glutamate Transporter 1 in the Rapid Antidepressant-Like Effect of Ketamine in Mice
Source: Front Behav Neurosci. 2022 Mar 2;16:789524. doi: 10.3389/fnbeh.2022.789524 (PMC8926310; doi:10.3389/fnbeh.2022.789524)
Supplement: Supplementary file 5 [file Data_Sheet_5.PDF]

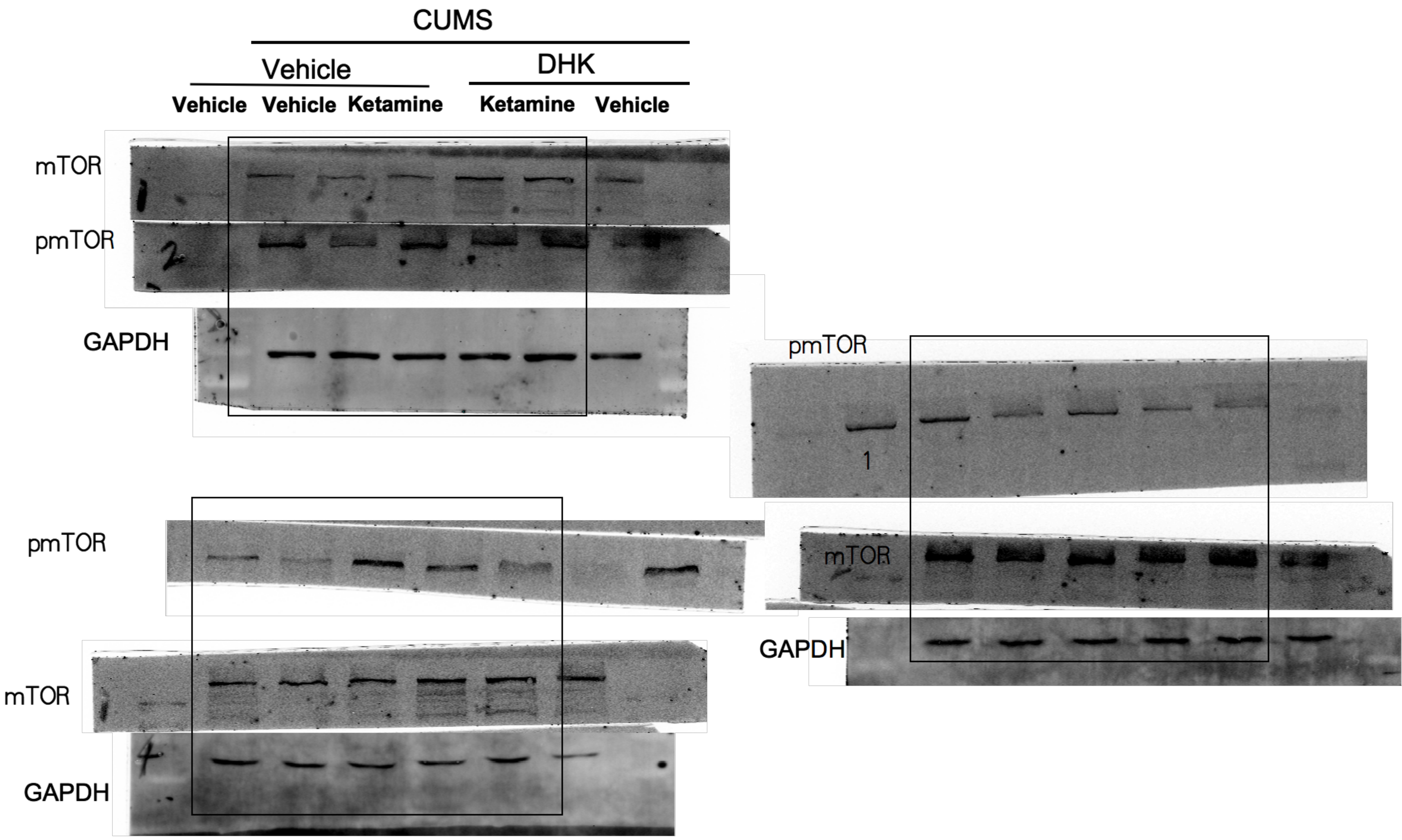
CUMS
Vehicle
DHK
Vehicle
Vehicle
Ketamine
Ketamine
Vehicle
mTOR
pmTOR
GAPDH
pmTOR
1
pmTOR
mTOR
mTOR
GAPDH
GAPDH
