# Supplementary material for: The Regulation of Glutamate Transporter 1 in the Rapid Antidepressant-Like Effect of Ketamine in Mice
Source: Front Behav Neurosci. 2022 Mar 2;16:789524. doi: 10.3389/fnbeh.2022.789524 (PMC8926310; doi:10.3389/fnbeh.2022.789524)
Supplement: Supplementary file 6 [file Data_Sheet_6.PDF]

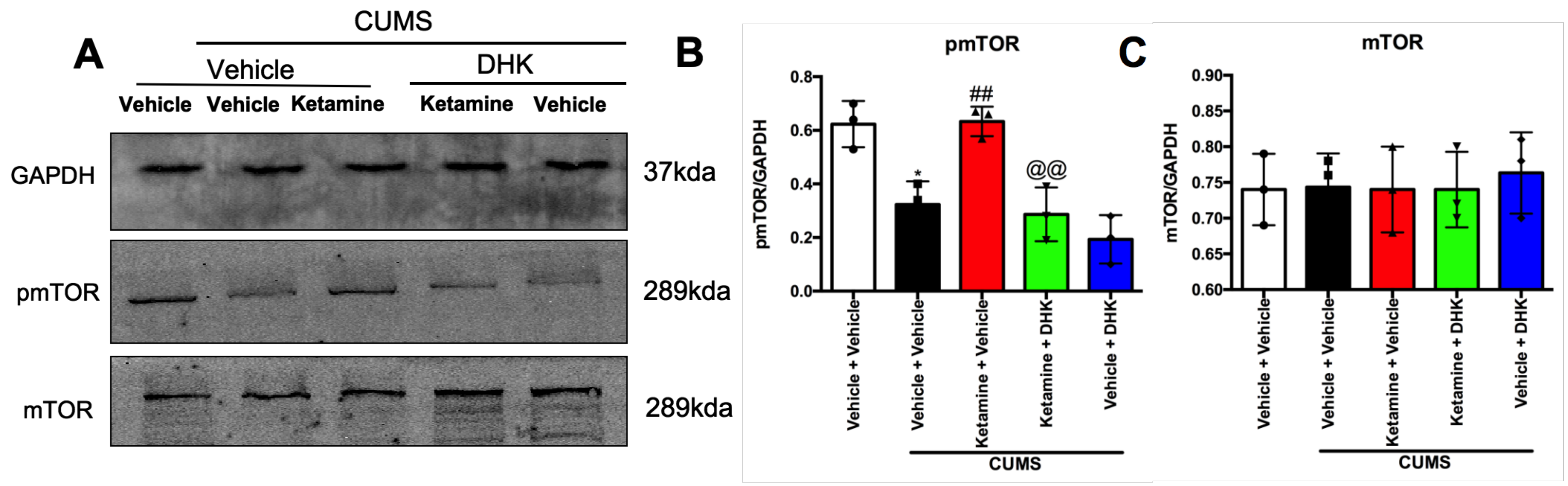

	Ordinary one-way ANOVA<br>ANOVA	A	B	C	D	E
		Data Set-A	Data Set-B	Data Set-C	Data Set-D	Data Set-E
		Y	Y	Y	Y	Y
1	Table Analyzed	pmTOR				
2						
3	ANOVA summary					
4	F	17.16				
5	P value	0.0002				
6	P value summary	***				
7	Are differences among means statistically significant? (P < 0.05)	Yes				
8	R square	0.8728				
9						
10	Brown-Forsythe test					
11	F (DFn, DFd)	0.1618 (4, 10)				
12	P value	0.9530				
13	P value summary	ns				
14	Significantly different standard deviations? (P < 0.05)	No				
15						
16	Bartlett's test					
17	Bartlett's statistic (corrected)					
18	P value					
19	P value summary					
20	Significantly different standard deviations? (P < 0.05)					
21						
22	ANOVA table	SS	DF	MS	F (DFn, DFd)	P value
23	Treatment (between columns)	0.4951	4	0.1238	F (4, 10) = 17.16	P = 0.0002
24	Residual (within columns)	0.07213	10	0.007213		
25	Total	0.5672	14			
26						
27	Model comparison	SS	DF	Probability it is correct		
28	Null H. All population means identical	0.5672	14	0.12%		
29	Alternative H: Distinct population means	0.07213	10	99.88%		
30	Ratio of probabilities			826.3		
31	Difference in AICc			13.43		
32						
33	Data summary					
34	Number of treatments (columns)	5				
35	Number of values (total)	15				

	Ordinary one-way ANOVA<br>ANOVA	A	B	C	D	E
		Data Set-A	Data Set-B	Data Set-C	Data Set-D	Data Set
		Y	Y	Y	Y	Y
1	Table Analyzed	mTOR				
2						
3	ANOVA summary					
4	F	0.1079				
5	P value	0.9770				
6	P value summary	ns				
7	Are differences among means statistically significant? (P < 0.05)	No				
8	R square	0.04137				
9						
10	Brown-Forsythe test					
11	F (DFn, DFd)	0.03234 (4, 10)				
12	P value	0.9976				
13	P value summary	ns				
14	Significantly different standard deviations? (P < 0.05)	No				
15						
16	Bartlett's test					
17	Bartlett's statistic (corrected)					
18	P value					
19	P value summary					
20	Significantly different standard deviations? (P < 0.05)					
21						
22	ANOVA table	SS	DF	MS	F (DFn, DFd)	P value
23	Treatment (between columns)	0.001240	4	0.0003100	F (4, 10) = 0.1079	P = 0.9770
24	Residual (within columns)	0.02873	10	0.002873		
25	Total	0.02997	14			
26						
27	Model comparison	SS	DF	Probability it is correct		
28	Null H. All population means identical	0.02997	14	99.98%		
29	Alternative H: Distinct population means	0.02873	10	0.02%		
30	Ratio of probabilities			4597		
31	Difference in AICc			-16.87		
32						
33	Data summary					
34	Number of treatments (columns)	5				
35	Number of values (total)	15				
