# Supplementary material for: The Regulation of Glutamate Transporter 1 in the Rapid Antidepressant-Like Effect of Ketamine in Mice
Source: Front Behav Neurosci. 2022 Mar 2;16:789524. doi: 10.3389/fnbeh.2022.789524 (PMC8926310; doi:10.3389/fnbeh.2022.789524)
Supplement: Supplementary file 7 [file Data_Sheet_7.PDF]

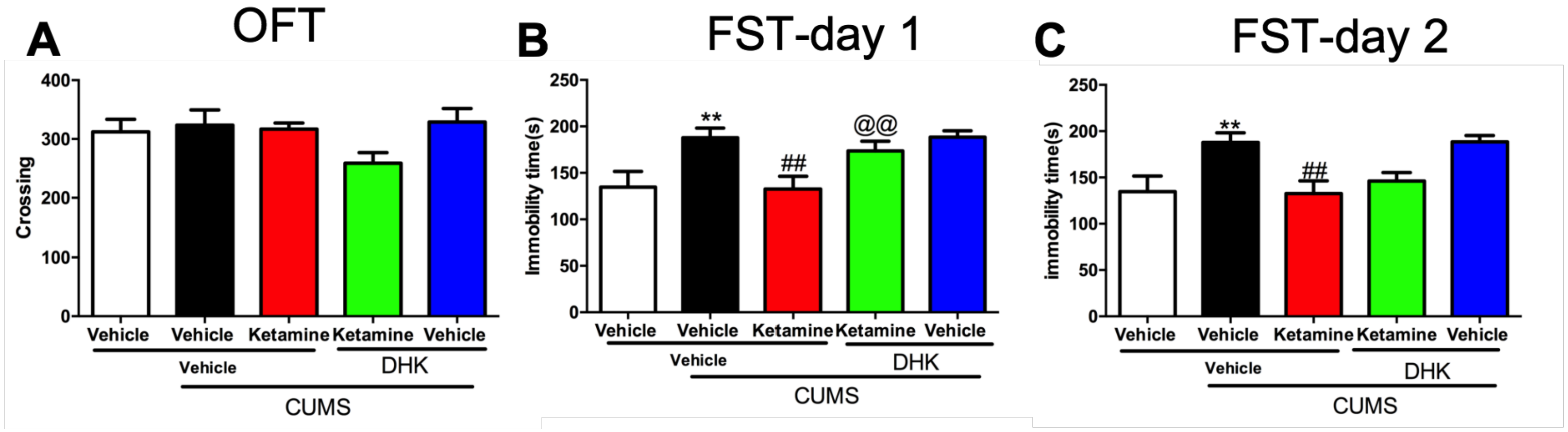

	Ordinary one-way ANOVA<br>ANOVA	A<br>Data Set-A	B<br>Data Set-B	C<br>Data Set-C	D<br>Data Set-D	E<br>Data Set-E
		Y	Y	Y	Y	Y
1	Table Analyzed	Data 1				
2						
3	ANOVA summary					
4	F	1.712				
5	P value	0.1616				
6	P value summary	ns				
7	Are differences among means statistically significant? (P <	No				
8	R square	0.1184				
9						
10	Brown-Forsythe test					
11	F (DFn, DFd)	1.609 (4, 51)				
12	P value	0.1863				
13	P value summary	ns				
14	Significantly different standard deviations? (P < 0.05)	No				
15						
16	Bartlett's test					
17	Bartlett's statistic (corrected)	8.606				
18	P value	0.0717				
19	P value summary	ns				
20	Significantly different standard deviations? (P < 0.05)	No				
21						
22	ANOVA table	SS	DF	MS	F (DFn, DFd)	P value
23	Treatment (between columns)	32987	4	8247	F (4, 51) = 1.712	P = 0.1616
24	Residual (within columns)	245602	51	4816		
25	Total	278588	55			
26						
27	Model comparison	SS	DF	Probability it is corre		
28	Null H. All population means identical	278588	55	77.12%		
29	Alternative H: Distinct population means	245602	51	22.88%		
30	Ratio of probabilities			3.371		
31	Difference in AICc			-2.431		
32						
33	Data summary					
34	Number of treatments (columns)	5				
35	Number of values (total)	56				

1	Table Analyzed	Data 1				
2						
3	ANOVA summary					
4	F	5.228				
5	P value	0.0013				
6	P value summary	**				
7	Are differences among means statistically significant? (P < 0.05)	Yes				
8	R square	0.2908				
9						
10	Brown-Forsythe test					
11	F (DFn, DFd)	3.030 (4, 51)				
12	P value	0.0257				
13	P value summary	*				
14	Significantly different standard deviations? (P < 0.05)	Yes				
15						
16	Bartlett's test					
17	Bartlett's statistic (corrected)	8.831				
18	P value	0.0655				
19	P value summary	ns				
20	Significantly different standard deviations? (P < 0.05)	No				
21						
22	ANOVA table	SS	DF	MS	F (DFn, DFd)	P value
23	Treatment (between columns)	35336	4	8834	F (4, 51) = 5.228	P = 0.0013
24	Residual (within columns)	86177	51	1690		
25	Total	121513	55			
26						
27	Model comparison	SS	DF	Probability it is correct		
28	Null H. All population means identical	121513	55	0.76%		
29	Alternative H: Distinct population means	86177	51	99.24%		
30	Ratio of probabilities			131.3		
31	Difference in AICc			9.755		
32						
33	Data summary					
34	Number of treatments (columns)	5				
35	Number of values (total)	56				

3	ANOVA summary					
4	F	5.447				
5	P value	0.0010				
6	P value summary	***				
7	Are differences among means statistically significant? (P < 0.05)	Yes				
8	R square	0.2993				
9						
10	Brown-Forsythe test					
11	F (DFn, DFd)	3.236 (4, 51)				
12	P value	0.0193				
13	P value summary	*				
14	Significantly different standard deviations? (P < 0.05)	Yes				
15						
16	Bartlett's test					
17	Bartlett's statistic (corrected)	9.859				
18	P value	0.0429				
19	P value summary	*				
20	Significantly different standard deviations? (P < 0.05)	Yes				
21						
22	ANOVA table	SS	DF	MS	F (DFn, DFd)	P value
23	Treatment (between columns)	35798	4	8950	F (4, 51) = 5.447	P = 0.0010
24	Residual (within columns)	83789	51	1643		
25	Total	119588	55			
26						
27	Model comparison	SS	DF	Probability it is correct		
28	Null H. All population means identical	119588	55	0.54%		
29	Alternative H: Distinct population means	83789	51	99.46%		
30	Ratio of probabilities			184.4		
31	Difference in AICc			10.43		
32						
33	Data summary					
34	Number of treatments (columns)	5				
35	Number of values (total)	56				
