# Supplementary material for: The Regulation of Glutamate Transporter 1 in the Rapid Antidepressant-Like Effect of Ketamine in Mice
Source: Front Behav Neurosci. 2022 Mar 2;16:789524. doi: 10.3389/fnbeh.2022.789524 (PMC8926310; doi:10.3389/fnbeh.2022.789524)
Supplement: Supplementary file 8 [file Data_Sheet_8.docx]

**Supplementary data**

**The regulation of glutamate transporter 1 (GLT1) in the rapid antidepressant-like effect of ketamine in mice**

Yaping Chen^1,2,3△^, Mengxin Shen^1^, Xu Liu^4△^, Jiangping Xu^2*^, Chuang Wang^1*^

1. Zhejiang Provincial Key Laboratory of Pathophysiology, Ningbo University School of Medicine, 818 Fenghua Road, Ningbo, Zhejiang 315211, China

2. School of Pharmaceutical Sciences, Southern Medical University, Guangzhou 510515, China.

3. College of Pharmacy, Fujian University of Traditional Chinses Medicine, Fuzhou 350108, China

4. Department of Pharmacy, General Hospital of Chinese People's Armed Police Forces, Beijing 100039, China.

△These authors contributed equally to this study.

*Correspondences: Chuang Wang, M.D., Ph.D.

E-mail: [wangchuang@nbu.edu.cn](mailto:wangchuang@nbu.edu.cn); wanglovechuang@163.com;

Jiangping Xu, M.D., Ph.D.

E-mail: [jpx@smu.edu.cn](mailto:jpx@smu.edu.cn)


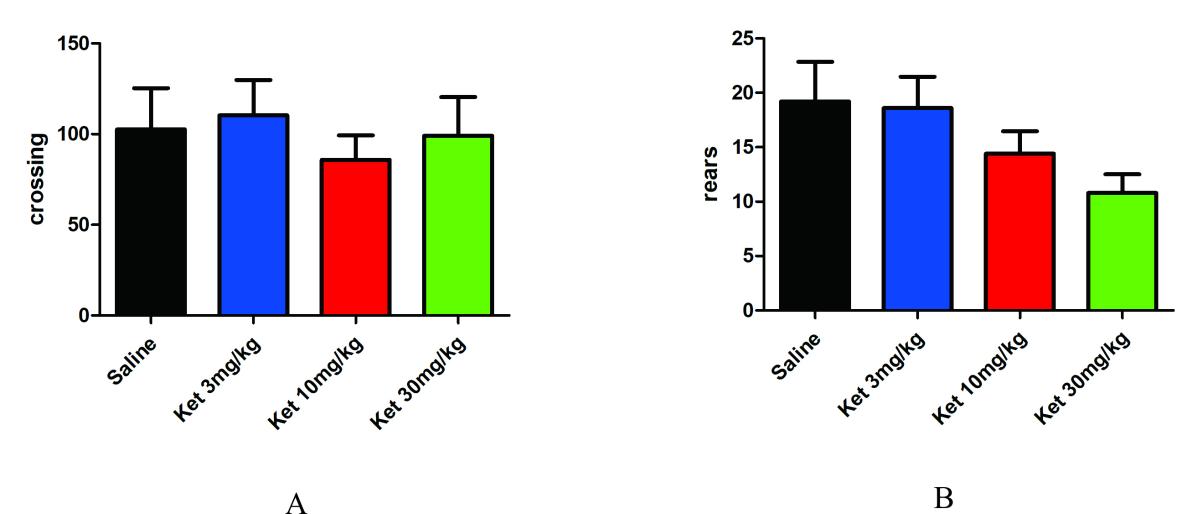


**Supplemental Figure 1. The effect of different doses of Ketamine in the OFT of mice.** The crossing score of mice (A), the rearing score of mice (B). The data are expressed as means ± SEM, n = 8.

**
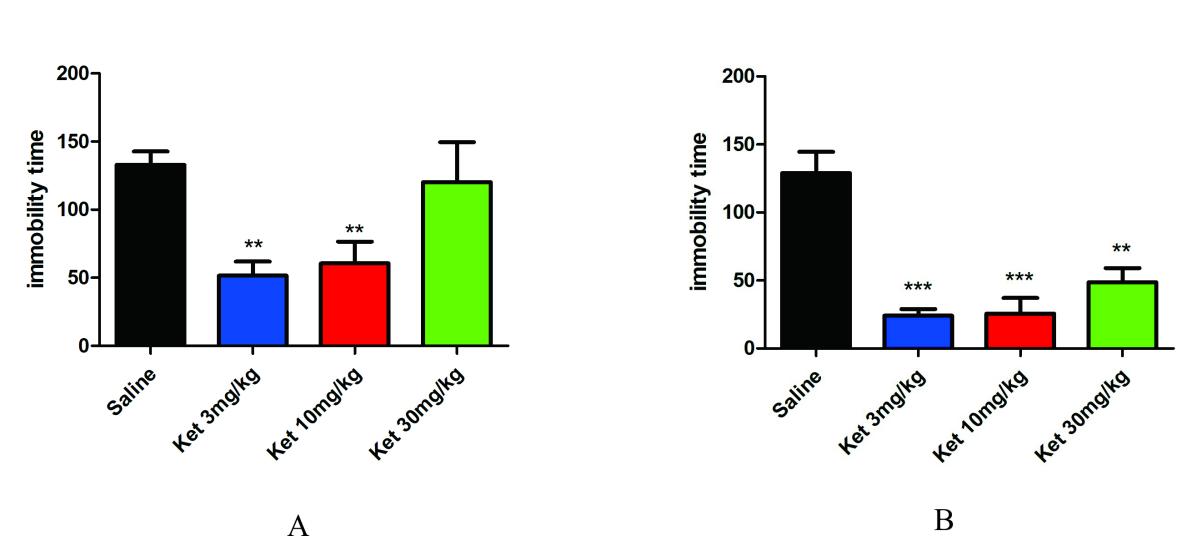
**

**Supplemental Figure 2. The effect of Ketamine on depression-like behaviors induced by CUMS.** The immobility time of mice in the FST (A) and TST (B). The data are expressed as means ± SEM, n = 8; *P < 0.05, **P < 0.01, ***P < 0.001 compared with the group deal with saline.

**Supplementary Figure 3. Single treatment with NBQX (microinjection) and verapamil (i.p.) had no effects alone in the OFT and FST of mice.** (A) The timeline of the drug treatment and behavioral tasks. (B) Line crossings in the OFT (NBQX, Student’s t test, t16=2.001, P = 0.0626; Verapamil, Student’s t test, t16=1.268, P = 0.2228); (C) Rearings in the OFT (NBQX, Student’s t test, t16=1.355, P = 0.1942; Verapamil, Student’s t test, t16=0.2598, P = 0.7983); (D) Immobility time in the FST (NBQX, Student’s t test, t16=0.8302, P = 0.4186; Verapamil, Student’s t test, t16=0.8337, P = 0.4167). The data are expressed as means ± S.E.M; n = 9/mice for behavioral test.
